# Supplementary material for: Adaptive radiofrequency shimming in MRI using reconfigurable dielectric materials
Source: Sci Rep. 2025 Dec 24;16:2758. doi: 10.1038/s41598-025-32636-0 (PMC12824193; doi:10.1038/s41598-025-32636-0)
Supplement: Supplementary file 1 — Supplementary Information. [file 41598_2025_32636_MOESM1_ESM.pdf]

Adaptive Radiofrequency Shimming in MRI using Reconfigurable Dielectric Materials: Supplementary Materials

Paulina Šiurytė, Robert van de Velde, Jasper van Leeuwen, Kadir Berat Yildirim, Ömer Can Akgün, Wyger Brink and Sebastian Weingärtner

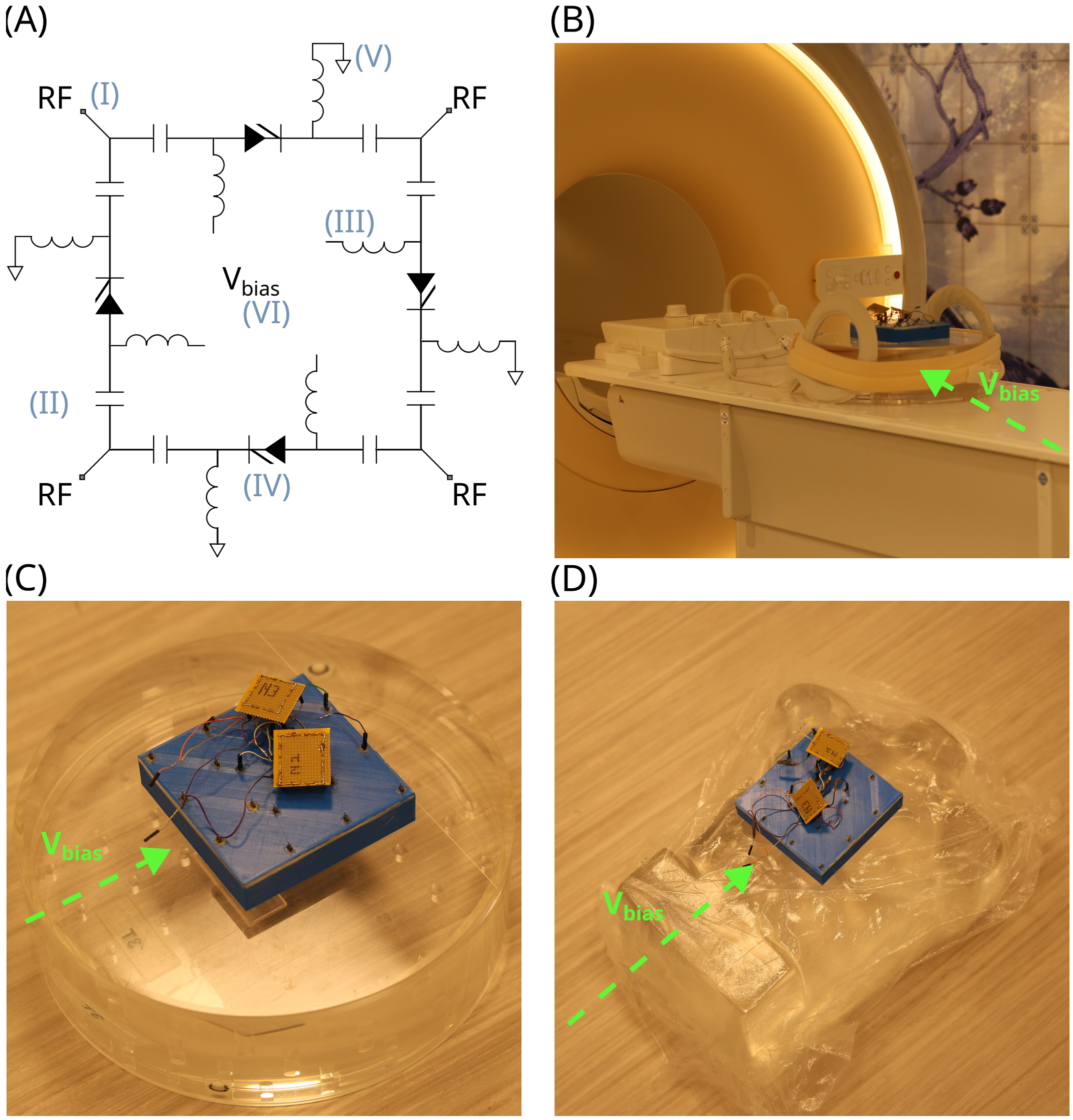

Figure S1 . RF switch experimental setup and circuit diagram. (A) Schematic diagram of the RF switching circuit for interconnecting four elements of the dielectric array. (I) Header pin connector. (II) DC choke. (III) RF choke. (IV) PIN diode. (V) Ground. (VI) Bias voltage (V_{bias}). (B) Cylindrical phantom and dielectric array setup in the scanner, with an indicated direction of bias voltage application. (C-D) A closer look at the orientation of the phantom and dielectric array.

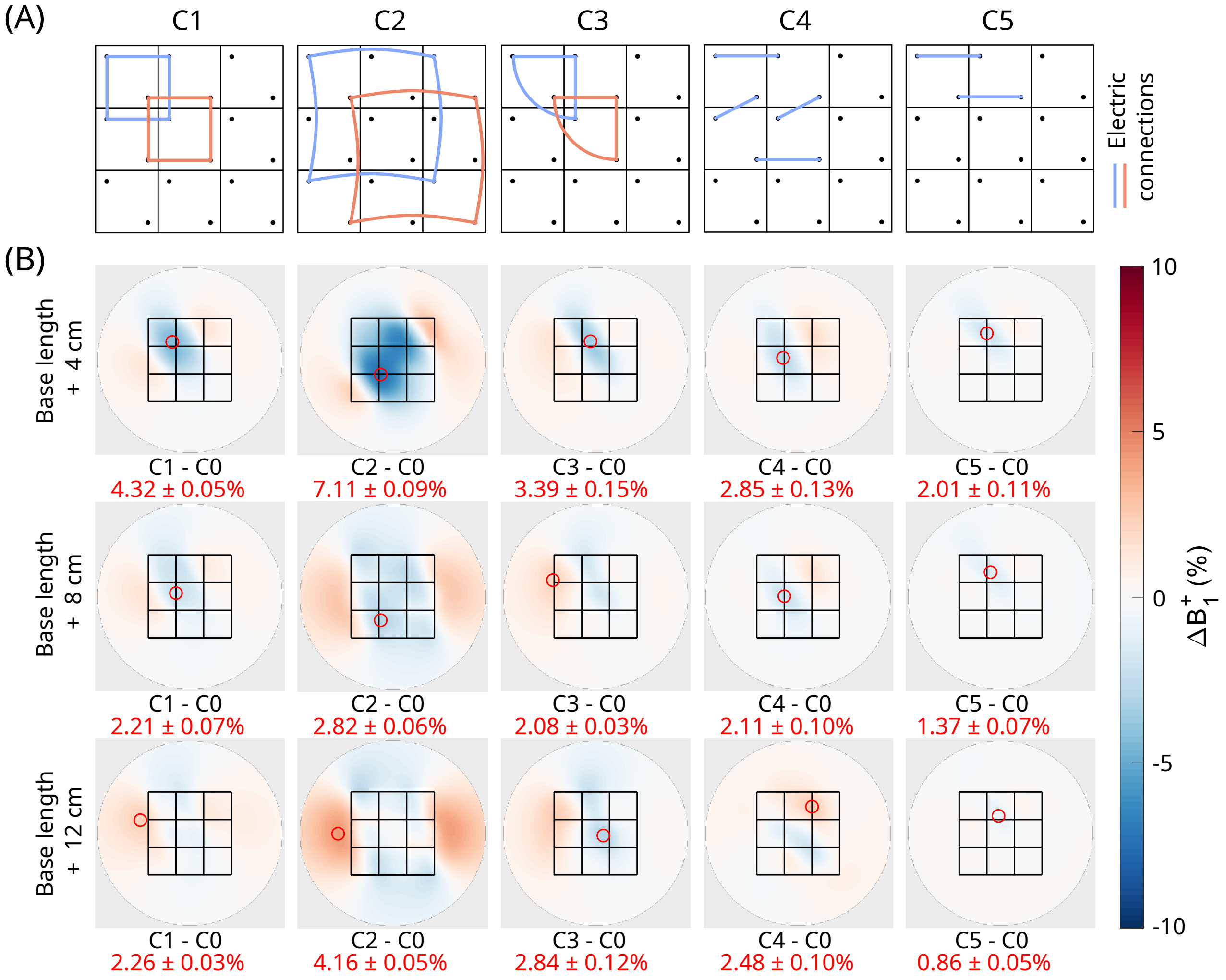

Figure S2 . Simulated B_1^+ modulation for several studied coupling configurations. (A) Schematic representation of a 3×3 array of dielectric pockets with different coupling configurations. C0 is uncoupled and is provided as a reference case. (B) B_1^+ difference maps evaluated at a depth of 15 mm inside the cylindrical phantom and normalized to 1000 W of stimulated power. Each subplot shows the difference in the normalized B_1^+ field of the coupled case (C1-C5) and the baseline C0. The outline of the cask is shown in black, and the considered ROIs are indicated by a red circle.

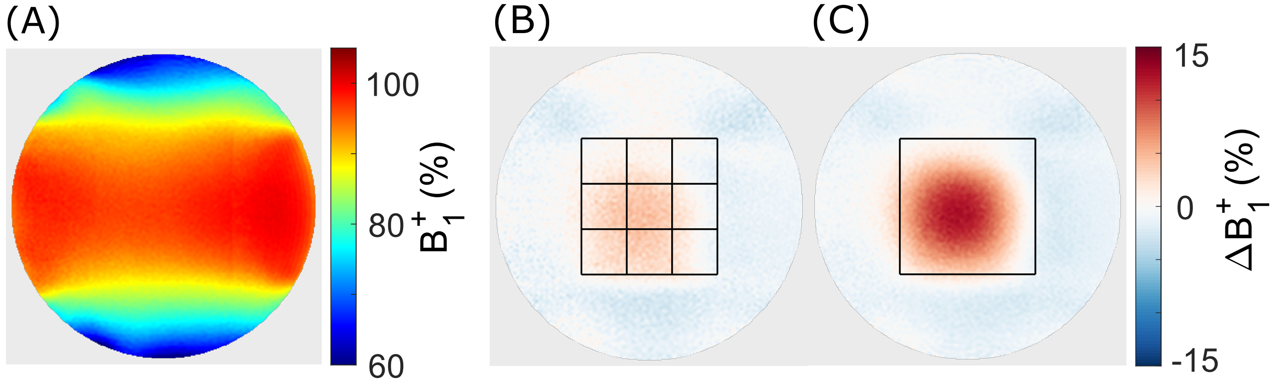

Figure S3 . (A) B_1^+ map in the disk-shaped phantom with no shimming device. (B) B_1^+ modulation with an unwired 3×3 dielectric array with respect to no device present on the phantom. (C) B_1^+ modulation with a homogeneous dielectric pad.
